# NOX, NOX Who is There? The Contribution of NADPH Oxidase One to Beta Cell Dysfunction

**DOI:** 10.3389/fendo.2013.00040

**Published:** 2013-04-03

**Authors:** David A. Taylor-Fishwick

**Affiliations:** ^1^Department of Internal Medicine, Strelitz Diabetes Center, Eastern Virginia Medical SchoolNorfolk, VA, USA; ^2^Department of Microbiology and Molecular Cell Biology, Eastern Virginia Medical SchoolNorfolk, VA, USA

**Keywords:** NADPH oxidase, reactive oxygen species, cytokines, beta cell dysfunction, src-kinase

## Abstract

Predictions of diabetes prevalence over the next decades warrant the aggressive discovery of new approaches to stop or reverse loss of functional beta cell mass. Beta cells are recognized to have a relatively high sensitivity to reactive oxygen species (ROS) and become dysfunctional under oxidative stress conditions. New discoveries have identified NADPH oxidases in beta cells as contributors to elevated cellular ROS. Reviewed are recent reports that evidence a role for NADPH oxidase-1 (NOX-1) in beta cell dysfunction. NOX-1 is stimulated by inflammatory cytokines that are elevated in diabetes. First, regulation of cytokine-stimulated NOX-1 expression has been linked to inflammatory lipid mediators derived from 12-lipoxygenase activity. For the first time in beta cells these data integrate distinct pathways associated with beta cell dysfunction. Second, regulation of NOX-1 in beta cells involves feed-forward control linked to elevated ROS and Src-kinase activation. This potentially results in unbridled ROS generation and identifies candidate targets for pharmacologic intervention. Third, consideration is provided of new, first-in-class, selective inhibitors of NOX-1. These compounds could have an important role in assessing a disruption of NOX-1/ROS signaling as a new approach to preserve and protect beta cell mass in diabetes.

## Beta Cell Dysfunction

Beta cell dysfunction and loss of functional beta cell mass is a principle contributor to the development of diagnosable diabetes. Current therapeutic options do little to halt or reverse a loss of beta cell function. There remains a clinical need to better understand the events contributing to diminished beta cell function and to develop new strategies for beta-cell preservation and protection. As projected by the Centers for Disease Control and Prevention, one in three adults in the United States of America could have diabetes by 2050 (Boyle et al., 2010). In children (less than 20 years of age), it is conservatively estimated that a 23% increase in type 1 diabetes and a 49% increase in type 2 diabetes will occur over the next 40 years. Worryingly, this prevalence could increase more than threefold with shifts in population demographics (Imperatore et al., 2012). Globally, a significant increase in worldwide diabetes prevalence is projected^1^.

## Oxidative Stress in the Beta Cell

Oxidative stress in the beta cell is recognized as a pathogenic step leading to loss of beta cell function (Lenzen, 2008). Pancreatic islets have been shown to express low activity of free-radical detoxifying enzymes (e.g., catalase, superoxide dismutase, glutathione peroxidase) when compared to other tissues (Grankvist et al., 1981; Lenzen et al., 1996; Tiedge et al., 1997; Modak et al., 2007). Islets are also very poor in rectifying oxidative damage to DNA (Modak et al., 2009). Thus, under conditions of sustained activation of intracellular reactive species, islets are readily overwhelmed and undergo oxidative stress (Lenzen, 2008). Under oxidative stress conditions, the elevated reactive oxygen species (ROS), in addition to oxidizing proteins, lipids, and DNA, also activate stress-sensitive second messengers such as p38MAPK, JNK (Purves et al., 2001), and PKC (Koya and King, 1998). A consequence of JNK activation is a translocation of the homeodomain transcription factor *pdx-1* from the nucleus to the cytoplasm. PDX-1 is a key transactivator of the insulin gene (Ohneda et al., 2000). As *pdx-1* also transactivates its own expression (Kawamori et al., 2003), the consequence of cytoplasmic translocation of *pdx-1* in conditions of oxidative stress further limits insulin expression and contributes to beta cell dysfunction. The beta cell therefore, has to orchestrate a delicate balance in ROS generation. While on one hand an overstimulation of ROS is destructive to beta cell function and survival, on the other hand a transient increase in ROS generation is a required second messenger for glucose-stimulated insulin secretion (Goldstein et al., 2005; Pi et al., 2007; Newsholme et al., 2009). Reinforcing this requirement, neutralization of ROS activity in beta cells with anti-oxidants decreases the glucose-stimulated insulin response (Morgan et al., 2009).

Serum conditions associated with the diabetic state, increased pro-inflammatory cytokines, high free fatty acids (FFA), and elevated glucose levels, are all potent inducers of elevated cellular ROS (Janciauskiene and Ahren, 2000; Oliveira et al., 2003; Cunningham et al., 2005; Inoguchi and Nawata, 2005; Nakayama et al., 2005; Uchizono et al., 2006; Morgan et al., 2007; Michalska et al., 2010). Inflammation and elevation in pro-inflammatory cytokines is an established feature of type 1 diabetes (Eizirik and Mandrup-Poulsen, 2001; Jorns et al., 2005), and in recent studies low-grade chronic inflammation and an increase in serum pro-inflammatory cytokines have been recognized as key features of type 2 diabetes (Catalan et al., 2007; Steinberg, 2007; Tilg and Moschen, 2008; Al-Maskari et al., 2010; Igoillo-Esteve et al., 2010; Kang et al., 2010; Su et al., 2010). Within the beta cell, cellular sources of ROS originate from induced mitochondrial stress (reviewed in Newsholme et al., 2007) and endoplasmic reticulum stress (reviewed in Volchuk and Ron, 2010). While these have been considered the main sources of ROS in pancreatic islets, identification of NADPH oxidase complexes in beta cells have brought the issue of the relative contribution to ROS generation under debate.

## NOX Family of NADPH Oxidases

**Figure 1:**
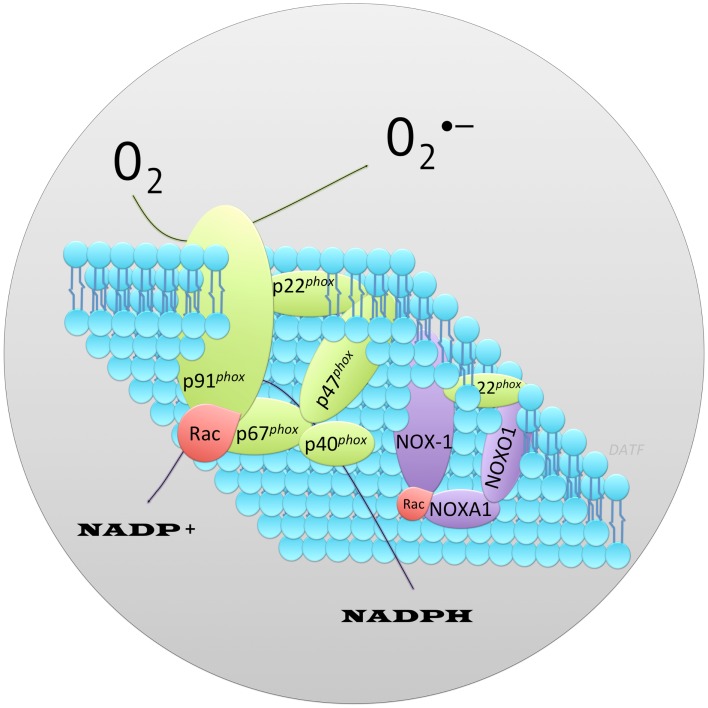
**Schematic representation of the protein components that form the archetype phagocyte NADPH oxidase, NOX-2 contrasted to NOX-1**. Illustrated is the core catalytic component of phagocyte NADPH oxidase, p91*^phox^* and, associated protein subunits required for a functional enzyme (shown in green). The widely-expressed membrane associated protein p22*^phox^* stabilizes the core catalytic component facilitating recruitment of cytosolic adaptor proteins (p67*^phox^*, p47*^phox^*, p40*^phox^*) and small GTPase, Rac (shown red). These are required for a functional oxidase. Superoxide (${\text{O}}_{2}^{∙-}$) is generated via a one electron reduction of oxygen by NADPH. By analogy, the functional NOX-1 enzyme consists of distinct protein subunits (shown in purple) in a complex with p22*^phox^* (green) and Rac (red). NOX-1, the core catalytic component, interacts with homologs of p47*^phox^* and p67*^phox^* called NOXO1 (NOX Organizer Protein 1) and NOXA1 (NOX Activator Protein 1) respectively.

NOX family of NADPH oxidases are proteins that transfer electrons across biological membranes (plasma or organelle). Their function is the generation of ROS, superoxide, and hydrogen peroxide (H_2_O_2_). The phagocyte NADPH oxidase was the first identified example of an enzyme system where ROS generation was the primary function rather than a byproduct, as seen in mitochondria and other cell components. Phagocyte NADPH oxidase function is best recognized in the respiratory (oxidative) burst response, which is a key component of innate immunity (Quinn and Gauss, 2004). Activation of phagocyte NADPH oxidase occurs through a complex series of protein interactions (Figure 1). The core catalytic component of NADPH oxidase, gp91*^phox^*, is stabilized in the membrane by p22*^phox^*. Recruitment of the adaptor protein p47*^phox^* facilitates addition to the complex of p40*^phox^*, p67*^phox^*, and Rac (small GTP-binding protein), the later two appear to regulate catalysis (Abo et al., 1991; Ando et al., 1992; Wientjes et al., 1993; Heyworth et al., 1994; Hordijk, 2006; Orient et al., 2007; Guichard et al., 2008; Sumimoto, 2008). Genome sequencing has identified a family of NOX proteins that form distinct NADPH oxidase complexes. Homologs of the core catalytic component subunit of phagocyte NADPH oxidase have also been identified (reviewed in Bedard and Krause, 2007, and termed NOX-1, -3,-4,-5, DUOX1-2). Under this nomenclature the core catalytic subunit of phagocyte NADPH oxidase (gp91*^phox^*) is termed NOX-2. In the beta cell, expression of NOX-1, NOX-2, and NOX-4 has been reported. With analogy to the phagocyte NADPH oxidase (NOX-2), the functional unit for NOX-1 is also dependent upon its association with cytosolic subunits. The subunits that bind NOX-1 are NOXO1 (NOX organizer 1) and NOXA1 (NOX activator 1), which while being distinct proteins are homologs of p47*^phox^* and p67*^phox^* respectively (Banfi et al., 2003; Geiszt et al., 2003a; Takeya et al., 2003; Cheng and Lambeth, 2004; Cheng et al., 2006). Reconstitution experiments have shown the functional unit for NOX-1 to require NOX-1/NOXO1/NOXA1 (Banfi et al., 2003; Geiszt et al., 2003a; Takeya et al., 2003; Cheng and Lambeth, 2004). Binding of p40*^phox^* or a homolog is not important for NOX-1 activity (Takeya et al., 2003). NOX-4 activity does not appear to require association with a NOX organizer/activator (Ambasta et al., 2004; Martyn et al., 2006). However, NOX-1 and NOX-4 activity requires association with p22*^phox^*, which is widely-expressed in tissues (Cheng et al., 2001), and Rac, which is expressed in beta cells, among other tissues (reviewed in Kowluru, 2010). Inhibition of Rac activation reduced oxidative stress mediated by high glucose and high FFA in INS-1 cells (Subasinghe et al., 2011). Expression of NOXO1 and NOXA1 has been reported in pancreas and beta cells (Uchizono et al., 2006). Thus, stimuli which upregulate NADPH oxidase core catalytic subunit expression in beta cells would be expected to promote the formation of functional complexes and increase the generation of ROS leading to cellular oxidative stress and/or ROS mediated signal transduction. This was demonstrated to be true for NOX-1. Recombinant overexpression of NOX-1 in NIH 3T3 cells resulted in elevated cellular ROS and induction of redox signaling (Go et al., 2004).

## Role of NOX in Beta Cell Dysfunction

Role of NOX in beta cell dysfunction is supported by the observation that several NOX isoforms are expressed in pancreatic beta cells including NOX-1, NOX-2, NOX-4, NOXO1, and NOXA1 (Oliveira et al., 2003; Nakayama et al., 2005; Lupi et al., 2006; Shao et al., 2006; Uchizono et al., 2006). Diabetes relevant serum conditions, including, increased pro-inflammatory cytokines, elevated FFA (palmitate), or high glucose, induce NADPH oxidase expression (Morgan et al., 2007). Moreover, expression of NOX isoforms are increased in animal models of type 2 diabetes (Nakayama et al., 2005). Conversely, global inhibition of NADPH oxidase conferred protection to beta cells exposed to cytokines or FFA (Michalska et al., 2010). NADPH oxidase activity has been linked to regulation of insulin secretion. Generation of ROS modulates glucose-stimulated insulin secretion; having a positive effect upon acute ROS activation but a negative effect upon chronic ROS activation (Pi et al., 2007; Morgan et al., 2009) and reviewed in Goldstein et al. (2005) and Newsholme et al. (2009). Global inhibition of NADPH oxidase indicated NOX activity is necessary to initiate the transient ROS increase required for glucose-stimulated insulin secretion. However, specific roles of each NOX isoform remain to be elucidated. This is, in part, due to a lack of isoform-specific inhibitors (reviewed in Lambeth et al., 2008). NOX-2 activity in beta cells has been reported linking transient ROS elevation to insulin secretion, or association with mitochondrial dysregulation (Syed et al., 2011a,b; Matti et al., 2012). Further, mice deficient in NOX-2 were protected against streptozotocin-induced beta cell destruction (Xiang et al., 2010). A pathological role for NOX-derived ROS in beta cells is suggested with a linkage of NOX-2 activity in beta cell dysfunction, induced by very low-density lipoprotein or free fatty acid (Yuan et al., 2010; Jiao et al., 2012). Study of the contribution of other NOX isoforms in beta cell function/dysfunction is active. Targeting the relative contributions of NOX family members has been approached in genetic depletion studies. Recent study of mice genetically deficient in each of the NOX core catalytic subunits has questioned the necessity of NOX for insulin secretion in response to glucose stimulation. Individual deletion of NOX-1, NOX-2, or NOX-4 did not abolish glucose-stimulated insulin secretion as was seen (Li et al., 2012), and previously reported (Uchizono et al., 2006), for the inhibitor of NOX activity diphenylene iodonium (DPI). The resolution of this discrepancy lies in the non-specific inhibitory action of DPI which, as a flavoenzyme inhibitor, disrupts other flavin-dependent enzymes in addition to NOX (Riganti et al., 2004). A caveat to the NOX-deletion approach is that some degree of functional redundancy provided by the remaining NOX is not eliminated, although compensatory upregulation of other NOXs in the targeted NOX deleted mice was not observed (Li et al., 2012). This study by Li et al. provides focus on basal NOX expression. An additional consideration is the upregulation of NOX enzymes in response to pathogenic stimuli. Study has been made of the induced expression of NOX enzymes in islets and beta cells following pro-inflammatory cytokine stimulation, a condition that promotes beta cell dysfunction (Weaver et al., 2012). As shown in Table 1, stimulation of human islets, mouse islets, or murine beta cell lines with a pro-inflammatory cytokine cocktail of TNFα, IL-1β, and IFNγ, preferentially induces the expression of NOX-1. It is therefore possible that the activity of NOX-1 may bean important component to beta cell pathogenesis. A decrease in cytokine-induced NOX-1 expression was observed in genetically modified mice that had a co-associated enhanced glucose tolerance and resistance to streptozotocin induced hyperglycemia (Chang et al., 2010).

**Table 1:** **Relative induction of NOX subunit expression in islets and beta cells following stimulation with pro-inflammatory cytokines**.

	NOX-1	NOX-2	NOX-4	NOXA1	NOX01
Human islets	+++	±	−	ND	ND
Mouse islets	+++	+	−	ND	ND
βTC-3	+++	ND	ND	ND	ND
INS-1	+++	+	−	+++	−

*ND, not determined*.

## Regulation of NOX-1 Expression

Regulation of NOX-1 expression has been reported by a variety of factors including angiotensin II (Suh et al., 1999; Lassegue et al., 2001; Wingler et al., 2001; Katsuyama et al., 2002), interferon (Geiszt et al., 2003b), the PKC activator PMA (Geiszt et al., 2003a; Takeya et al., 2003), and epidermal growth factor receptor (EGFR) activation involving PI3K and PKC (Fan et al., 2005). The promoter region for NOX-1 contains binding elements for AP-1, NFκB, CREB, STATs, and Interferon (Kuwano et al., 2006). NOX-1 expression is enhanced by IFNγ activation of STAT1 (Kuwano et al., 2006) through binding of a γ-activated sequence (GAS) element located −3818 to −3810 bp (Kuwano et al., 2006). Binding sites for GATA transcription factors are also present (Brewer et al., 2006). Regulation of NOX-1 also is reported to involve an autoregulatory feedback loop that promotes its own upregulation (Sancho and Fabregat, 2010). This feedback is mediated through the upregulation of Src and ERK activity (Fan et al., 2005; Adachi et al., 2008; Sancho and Fabregat, 2010). Induction of NOX-1 in beta cells was recently linked to 12-lipoxygenase activity (Weaver et al., 2012). Lipoxygenases catalyze the oxygenation of cellular polyunsaturated fatty acids to form lipid inflammatory mediators. In the case of 12-lipoxygenase, arachidonic acid is converted to 12-hydroperoxyeicosatetraenoic acid (12HPETE), which is subsequently reduced to the more stable 12-hydroxyeicosatetraenoic acid (12-HETE) by glutathione peroxidase (Yamamoto et al., 1997; Brash, 1999). The role of 12-LO in inflammation, beta cells, and other physiologic and pathologic systems has been reviewed (Dobrian et al., 2011). With regard beta cell dysfunction and diabetes, 12-lipoxygenase has been established as a key mediator. Deletion of 12-LO in mice confers resistant to diabetes induced by low dose streptozotocin (Bleich et al., 1999) and blocks conversion to spontaneous diabetes in the NOD model of type 1 diabetes (McDuffie et al., 2008). A product of 12-LO activity, 12-HETE, has been reported in rat islets (Metz et al., 1983; Metz, 1985; Turk et al., 1985; Shannon et al., 1992; Kawajiri et al., 2000; Prasad et al., 2003), human islets (Chen et al., 2005; Persaud et al., 2007; Ma et al., 2010), and rodent beta cell lines (Bleich et al., 1995; Chen et al., 2005). Overexpression of 12-LO in beta cells leads to loss of function and activation of apoptosis (Prasad et al., 2003). Further, direct addition of 12-HETE impairs insulin secretion from beta cells, and can induce apoptosis (Chen et al., 2005). Pro-inflammatory cytokines induce 12-LO activity in islets and beta-cell lines (Bleich et al., 1995; Chen et al., 2005). Direct addition of 12-HETE or pro-inflammatory cytokines to beta cells induced NOX-1 expression (Weaver et al., 2012). Furthermore, inhibition of 12-LO activity, with selective small molecules (Kenyon et al., 2011), blocked the induction of NOX-1 by pro-inflammatory cytokines (Weaver et al., 2012). These data indicate an integration of pro-inflammatory cytokine stimulation and activation of 12-LO in the upregulation of NOX-1 in beta cells; a pathway linked to beta cell dysfunction.

## Redox Signaling

Redox signaling is a functional consequence of NOX upregulation. Over expression of NOX-1 in NIH 3T3 cells results in an upregulation of cellular ROS (Go et al., 2004). The capacity of NOX to elevate ROS production in beta cells has strikingly been reported to equal levels found in neutrophils (Oliveira et al., 2003). Both rat neutrophils and rat beta cells reach similar maximum levels of superoxide and H_2_O_2_ in response to a glucose challenge, albeit with different kinetics, 50% Vmax occurring at 5 and 45 min in neutrophils and beta cells respectively (Oliveira et al., 2003). While elevated cellular ROS readily brings to mind concepts of oxidative stress and a deleterious oxidizing environment, designed signaling cascades of oxidation-sensitive second messenger targets also result. Unlike the ROS products that result from several metabolic processes, the cellular function of NOX enzymes is to generate ROS. This suggests the option that target-specific signaling pathways may be regulated by low levels of ROS; distinct from a global generalized oxidative environment. Several signaling pathways are linked to a ROS-mediate regulation (reviewed in Goldstein et al., 2005; Mittler et al., 2011). These include activation of kinases in the mitogen activated protein (MAP) Kinase family and Src-kinase (Giannoni et al., 2005). Over expression of NOX-1 resulted in a concomitant activation of the MAP kinases, JNK, and ERK1/2 (Go et al., 2004). Other second messengers activated by NOX-1 include p38MAPK and AKT (Sancho and Fabregat, 2010). Thus, elevated expression of NOX-1, as occurs in beta cells upon exposure to elevated pro-inflammatory cytokines (Weaver et al., 2012), results in increased ROS expression that could induce discrete second messenger signaling, global destructive oxidative stress, or both.

## Regulation of NOX-1 Expression by ROS-Induced Second Messengers

**Figure 2:**
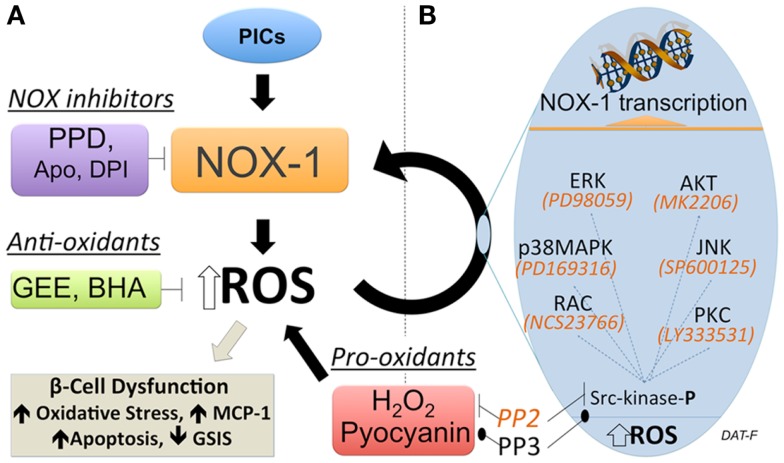
**Feed-forward regulation of NOX-1 expression in beta cells**. **(A)** Experimental approach to demonstrate a feed-forward regulation of NOX-1 in beta cells that involves NOX activity and cellular redox state. Inhibition of NADPH oxidase activity (purple box) with either a selective NOX-1/4 pyrazolopyridine dione inhibitor (PPD) or general inhibitors apocynin (Apo), diphenylene iodonium (DPI) blocks NOX-1 gene and protein expression initiated by pro-inflammatory cytokine (PIC) stimulation. This suggests NADPH oxidase activity regulates NOX-1 expression. Neutralization of ROS with general anti-oxidants (green box) glutathione-ethyl ester (GEE) or butylated hydroxyanisole (BHA) inhibited PIC-stimulated NOX-1. Conversely, direct elevation of ROS with pro-oxidants (red box) hydrogen peroxide (H_2_O_2_) or pyocyanin, upregulated NOX-1 expression. This places ROS elevation in a pathway regulating NOX-1 expression. Interruption of Src-kinase signaling with inhibitor PP2 blocked NOX-1 expression induced by pro-oxidant or PIC-stimulation. Interruption was not observed using the control (inactive) compound PP3. This indicates that activation of Src-kinase signaling that leads to NOX-1 gene transcription is a consequence of elevated ROS. Beta cell dysfunction, as measured by increase in apoptosis and loss of glucose-stimulated-insulin-secretion (GSIS), follows sustained elevation of ROS. **(B)** Candidate effectors of Src-kinase activation are shown along with selective inhibitors (italics), which are available to map key contributing pathways. In terms of disease pathology, self-sustaining NOX-1 would lead to unbridled ROS generation, oxidative stress, and induce beta cell dysfunction/destruction. Consequently, this pathway has potential for high impact therapeutic intervention.

Regulation of NOX-1 expression by ROS-induced second messengers has been proposed in the liver cell line FaO (Sancho and Fabregat, 2010). These data suggest a positive feed-forward regulation of NOX-1. In beta cells, which have a low anti-oxidant capacity relative to metabolic activity, a feed-forward control of ROS production could rapidly result in a pathological state. Consequently, identifying and inhibiting such regulation could prove important in developing new strategies for preservation and protection of functional beta cell mass in diabetes. Studies have been performed to address if regulation of NOX-1 in beta cells is subject to feed-forward control (Weaver and Taylor-Fishwick, 2013). A summary of the approach used is shown in Figure 2. These studies have driven the proposed model that while NOX-1 is induced by pro-inflammatory cytokine stimulation in beta cells, a component of NOX-1 expression derives from a self-sustained upregulation mediated by elevated ROS expression and second messengers. The first clue for a feed-forward regulation of NOX-1 in beta cells stemmed from the observation that the induced expression of NOX-1 in pro-inflammatory cytokine-stimulated beta cells was abrogated with inhibitors of NADPH oxidase activity. Since NOX activity is subsequent to induced gene expression, the simplest explanation of the data is that NOX activity upregulates NOX-1 gene expression, a feed-forward control. NOX-1 induction of ROS was further implicated. Addition of general anti-oxidants, which neutralize cellular ROS, inhibited NOX-1 expression induced from pro-inflammatory cytokine stimulation. Moreover pro-oxidants, that directly elevate cellular ROS in the absence of other stimuli, induced NOX-1 expression. The redox sensitive kinase Src-kinase was shown to be activated by ROS in this pathway and a selective inhibitor of Src-kinase, PP2, disrupted stimuli-induced NOX-1 expression. The disruption in NOX-1 was not observed with the inactive structural analog, PP3. Importantly, inhibitors of these resolved signaling pathways also protected beta cells from the damaging effects induced by pro-inflammatory cytokine stimulation (Weaver and Taylor-Fishwick, 2013). Pro-inflammatory cytokine simulation of beta cells leads to increased expression of MCP-1, loss of glucose-stimulated insulin secretion, and induction or apoptosis. These three readouts of beta cell function were preserved by disruption of the NOX-1 feed-forward regulation.

## Chemical Inhibition of NADPH Oxidase Activity

Chemical inhibition of NADPH oxidase activity is an active area of investigation. Historically accepted inhibitors of NADPH oxidase activity, such as apocynin and DPI have been acknowledged as contributing to misleading interpretations due to low enzyme selectivity (Riganti et al., 2004; Vejrazka et al., 2005; Heumuller et al., 2008; Castor et al., 2010). Apocynin (4′-hydroxy-3′methoxyacetophenone) is a naturally occurring methoxy-substituted catechol. First identified in the 1800s, apocynin was recognized, accepted, and widely used as a selective NADPH oxidase inhibitor from mid 1990. Though apocynin continues to be used and marketed as an inhibitor of NADPH oxidase, it is not considered selective for NOX subunits. Apocynin is a pro-drug activated by peroxidase, its specificity of action is questioned following reports of activity in peroxide-deficient cells. Further, apocynin stimulated ROS production in non-phagocytes (Vejrazka et al., 2005) and demonstrated anti-oxidant effects in endothelial and vascular smooth muscle cells (Heumuller et al., 2008). Likewise, DPI has been extensively described in research literature as a NADPH oxidase inhibitor. DPI inhibits electron transporters. As an inhibitor of flavoenzymes, DPI interrupts NADPH oxidase activity but also other flavin-dependent enzymes (Riganti et al., 2004). Thus, DPI is not a selective NADPH oxidase inhibitor. Reflective of the significant interest in NADPH oxidases and disease pathology, aggressive investigation for new and selective small molecular weight inhibitors of NOX subunits is ongoing (reviewed in Lambeth et al., 2008; Jaquet et al., 2009; Cifuentes-Pagano et al., 2012). With regards NOX-1, high throughput screen approaches have identified two compound series with selectivity over NOX-2 activity. Several pyrazolopyridine dione inhibitors with nanomolar potency to NOX-1 and NOX-4 have been described that exhibit ten-fold selectivity over NOX-2 inhibition (Laleu et al., 2010). The lead compound in this series, GKT 136901 is orally bioavailable. A second approach has identified an active phenothiazine (2-acetylphenothiazine, ML171) with nanomolar IC_50_ and 20-fold selectivity over NOX-2, and NOX-4 (Gianni et al., 2010). These compounds provide promise to explore the role of NOX subunits in disease pathology and assessment of their inhibition as new therapeutic approaches to disease.

In summary, the free-radical generating enzymes NADPH oxidases may prove to have a greater importance than previously recognized in pathogenic stimuli resulting in beta cell dysfunction. This is reflected by evidence of a feed-forward regulation of NOX-1 expression that could result in a self-sustaining and escalating cellular ROS expression, a potentially pathological state for which the beta cell is poorly prepared to rectify. While a newly emerging field, confidence in the contribution of NOX-1 to beta cell dysfunction will be achieved by pathway resolution. Selective chemical inhibition of NOX-1 and description of the second messenger events that mediate ROS induction of NOX-1 in beta cells will facilitate pathway resolution. Candidate messengers and their inhibitors are shown in Figure 2B. Targeted disruption of a feed-forward regulation of NOX-1 in beta cells holds promise to help preserve and protect functional beta cell mass in diabetes. Clearly, old-generation inhibitors that have been proven to exhibit low specificity have resulted in missteps.

NOX, NOX

Who’s there?

Snaf

….?

Future development of safe, highly characterized, and selective NOX inhibitors is required. Considerable research is being directed at this aim and with the benefit of hindsight, a promise of future targets for new therapeutic strategies is anticipated.

## Conflict of Interest Statement

The authors declare that the research was conducted in the absence of any commercial or financial relationships that could be construed as a potential conflict of interest.

## Footnotes

^1^International Diabetes Federation (http://www.idf.org/diabetesatlas/5e/the-global-burden).
